# Effects of Fungicide and Adjuvant Sprays on Nesting Behavior in Two Managed Solitary Bees, *Osmia lignaria* and *Megachile rotundata*

**DOI:** 10.1371/journal.pone.0135688

**Published:** 2015-08-14

**Authors:** Derek R. Artz, Theresa L. Pitts-Singer

**Affiliations:** USDA-Agricultural Research Service, Pollinating Insect Research Unit, Logan, Utah, United States of America; French National Institute for Agricultural Research (INRA), FRANCE

## Abstract

There is a growing body of empirical evidence showing that wild and managed bees are negatively impacted by various pesticides that are applied in agroecosystems around the world. The lethal and sublethal effects of two widely used fungicides and one adjuvant were assessed in cage studies in California on blue orchard bees, *Osmia lignaria*, and in cage studies in Utah on alfalfa leafcutting bees, *Megachile rotundata*. The fungicides tested were Rovral 4F (iprodione) and Pristine (mixture of pyraclostrobin + boscalid), and the adjuvant tested was N-90, a non-ionic wetting agent (90% polyethoxylated nonylphenol) added to certain tank mixtures of fungicides to improve the distribution and contact of sprays to plants. In separate trials, we erected screened cages and released 20 paint-marked females plus 30–50 males per cage to document the behavior of nesting bees under treated and control conditions. For all females in each cage, we recorded pollen-collecting trip times, nest substrate-collecting trip times (i.e., mud for *O*. *lignaria* and cut leaf pieces for *M*. *rotundata*), cell production rate, and the number of attempts each female made to enter her own or to enter other nest entrances upon returning from a foraging trip. No lethal effects of treatments were observed on adults, nor were there effects on time spent foraging for pollen and nest substrates and on cell production rate. However, Rovral 4F, Pristine, and N-90 disrupted the nest recognition abilities of *O*. *lignaria* females. Pristine, N-90, and Pristine + N-90 disrupted nest recognition ability of *M*. *rotundata* females. Electroantennogram responses of antennae of *O*. *lignaria* females maintained in the laboratory did not differ significantly between the fungicide-exposed and control bees. Our results provide the first empirical evidence that two commonly used fungicides and a non-ionic adjuvant can disrupt nest recognition in two managed solitary bee species.

## Introduction

Wild and managed pollinators play a vital role in ecosystem health and provide pollination services to many economically important crops worldwide that contribute to crop productivity and global food security [1–4]. Recently, however, declines in both managed and wild bee populations have been described in the United States and in Europe, noting that the spread of pathogens, loss of pollen host plants, use of pesticides, and habitat destruction and fragmentation among others as some of the potential causes for the declines [5–8]. For example, the number of European honey bee *Apis mellifera* L. (Apidae) colonies, the most widely used pollinating bee of agricultural crops, has experienced an alarming decline in North America and many European countries [9–12]. These colony losses have been attributed to a suite of factors including parasitic mites, viruses, fungal and bacterial diseases, and pesticides, all of which may contribute to the condition called Colony Collapse Disorder (CCD). While a complete understanding of CCD is yet unresolved, it is becoming clear that various chemical pesticides, particularly neonicotinoid insecticides, could be directly or indirectly linked to CCD [13–20].

The impacts and risks of wide-scale use of fungicides, systemic insecticides, and other pesticides on ecosystem functioning, including pollination, and the potential loss or declines in insect pollinator populations worldwide could adversely affect global biodiversity and human welfare [21,22]. Beyond an increase in bee mortality due to direct lethal doses of pesticides, sublethal effects are known. Pesticide-induced increases in susceptibility to pathogen infection can lead to changes in honey bee and bumble bee behaviors such as navigation and orientation, memory and learning, and foraging and feeding, all of which may reduce fecundity and overall colony fitness [23–28]. Yang et al. [29] found that honey bees orally exposed to a low dose of the neonicotinoid imidacloprid had increased foraging times and were delayed in their return trips to the hive, and at a much higher dose, had difficulty returning to their feeding site and disappeared altogether. Another study that exposed honey bees to the neonicotinoid thiamethoxam also indirectly caused high mortality when foragers failed to return to hives [27]. Colonies of the bumble bee *Bombus terrestris* (L.) (Apidae) exposed to field-realistic levels of imidacloprid had a significant drop in growth rate and an 85% reduction in new queen production at the end of the season compared to control colonies [30]. Field-level, chronic exposure to imidacloprid impaired *B*. *terrestris* foraging activity and increased worker mortality, causing a decrease in colony success [31,32].

Recently, Bernauer et al. [33] demonstrated that colonies of the bumble bee *B*. *impatiens* exposed to field-relevant levels of the fungicide chlorothalonil produced fewer workers and smaller-massed mother queens compared to control colonies in caged settings. Moreover, some combinations of fungicides with other chemicals in tank mixes have produced synergistic effects, thereby enhancing the toxicity of the chemical constituents [34–38]. Biddinger et al. [39] demonstrated that fenbuconazole, the active ingredient in several fungicides, when combined with the neonicotinoids acetamiprid and imidacloprid, was more toxic than either neonicotinoid product alone to *A*. *mellifera* and to Japanese hornfaced bees, *Osmia cornifrons* (Radoszkowski) (Megachilidae). Iwasa et al. [40] also demonstrated that combining certain neonicotinoids with certain fungicides increased the toxicity of neonicotinoids to honey bees. For instance, the toxicity of acetamiprid increased 244- and 105-fold when combined with the fungicides triflumizole and propiconazole, respectively.

Environmental exposure to insecticides and fungicides often occur together in certain agroecosystems and can have deleterious effects on bee health and immunity even at low levels. Pettis et al. [41] analyzed pollen samples collected from commercial *A*. *mellifera* hives in seven crops and found fungicides present in all 19 pollen samples. Healthy *A*. *mellifera* workers fed pesticide-laden pollen subsequently had reduced ability to resist infection by *Nosema ceranae*. Furthermore, high levels of fungicides in stored pollen have been shown to kill or inhibit the growth of certain beneficial microbes that are necessary to convert pollen into bee bread [42], suggesting that colony growth may slow due to impeded *A*. *mellifera* larval development. Fungicides detected in Belgian *A*. *mellifera* colonies were also positively associated with the probability of a colony showing disorders, which in turn could weaken the colonies [12].

Although there is growing global concern about declining bee populations and links to pesticide exposure, pesticide toxicity trials and risk assessment studies have focused mainly on *A*. *mellifera* and various *Bombus* species [15,20,38,43–46]. Only recently have studies examined the effects of fungicides and fungicide + insecticide and/or adjuvant mixtures on managed solitary bees, such as commercially used blue orchard bees *Osmia lignaria* Say (Megachilidae), red mason bees *O*. *bicornis* L., *O*. *cornifrons*, and alfalfa leafcutting bees *Megachile rotundata* (F.) (Megachilidae) [36,39,47–54]. *O*. *lignaria* is noted to be an excellent pollinator of fruit and nut trees such as almond, cherry, and apple [55–59]. *M*. *rotundata* is the primary pollinator for alfalfa seed production in the United States and Canada [60,61].

This study was initiated in response to reports from orchardists, pollination service providers, and bee biologists who observed that nesting *O*. *lignaria* females became inactive or abandoned their nests altogether after fungicide sprays, particularly in California cherry and almond orchards. Our objectives were to 1) detect any lethal and sublethal effects of two widely used fungicides, Rovral 4F (iprodione) and Pristine (pyraclostrobin + boscalid), and one adjuvant N-90 (90% polyethoxylated nonylphenol) on *O*. *lignaria* in cage settings in California; 2) similarly assess the effects of Pristine and Pristine + N-90 adjuvant on the nesting behavior of *M*. *rotundata* in Utah, and 3) explore one possible physiological mechanism for the disruption of normal behavior using an electroantennogram (EAG) to look for direct effects on antennal responses after fungicide exposure in *O*. *lignaria*.

## Materials and Methods

Fungicides used were technical grade Rovral 4F (Bayer CropScience) and Pristine (BASF). Rovral is a flowable fungicide containing 41.6% active ingredient (ai) iprodione and is registered for the control of various pathogens on tree fruit and nut crops, vegetables, and berry crops [62,63]. Iprodione is a dicarboximide fungicide that inhibits DNA and RNA synthesis and cell division in fungi [63,64]. Commercial-formulation Pristine (BASF) is a combination of two fungicides-12.8% ai pyraclostrobin and 25.2% ai boscalid. Pyraclostrobin is a strobilurin fungicide and boscalid is a carboxamide fungicide, both of which has different sites of action, but together targets all major stages of the fungal life cycle such as spore germination, germ tube elongation, mycelial growth, and sporulation via inhibition of mitochondrial respiration [65–68]. The adjuvant used in this study was N-90 (Grow More, Gardena, California), which is non-ionic and contains 90% polyethoxylated nonylphenol. Adjuvants are substances added to certain tank mixtures of fungicides to improve the performance or activity of the fungicide, and can also enhance the contact of spray solutions to leaf surfaces. Fungicide and adjuvant treatments were applied by hand-held sprayers indiscriminately to all plant structures and surfaces (e.g., leaves, flowers, etc.). Spray applications were made at night when bees were not active and were applied at the highest recommended field rates: Rovral 4F at 2.2 kg/ha; Pristine at 1.6 kg/ha; and N-90, N-90 adjuvant at 0.4 kg/ha. After one week of bee nesting in all cages on available forage (Week 1), sprays were applied to single cages in Weeks 2 and 3 according to Tables 1 and 2. Our main goals were to observe *O*. *lignaria* and *M*. *rotundata* females in field cage settings to detect adult lethality or sublethal effects on their foraging and nesting behaviors due to fungicide and adjuvant treatments.

**Table 1:** Fungicide and adjuvant spray treatments were applied weekly on planted fields of *Phacelia tanacetifolia* and *Sinapis alba* in screened field cages where 20 uniquely paint-marked *Osmia lignaria* females were nesting. Treatment sprays occurred on the evening of days 7 (April 13) and 14 (April 20) when bees were not active. The cage experiment was conducted at The Wonderful Company in Lost Hills, California in 2011.

Treatment Week 1, Week 2, Week 3		Application regime (date of spray)			Application rate (kg/ha)	
	Cage	Week 1 (Days 0–7)	Week 2 (Days 8–14)	Week 3 (Days 15–21)	Week 2	Week 3
NS*, Water**, Water	1	NS	Water	Water	—	—
NS, N-90, N-90	2	NS	N-90	N-90	0.4	0.4
NS, Rovral 4F, Rovral 4F	3	NS	Rovral 4F	Rovral 4F	2.2	2.2
NS, Pristine, Pristine	4	NS	Pristine	Pristine	1.6	1.6
NS, Rovral 4F, Pristine	5	NS	Rovral 4F	Pristine	2.2	1.6
NS, Pristine, Rovral 4F	6	NS	Pristine	Rovral 4F	1.6	2.2

* NS = no spray; nest initiation period to document normal nesting behavior

** Water was sprayed at equal volumes to treatment sprays

**Table 2:** Fungicides and adjuvant spray treatments and application rates applied weekly on planted fields of *Phacelia tanacetifolia* in screened field cages where 20 uniquely paint-marked *Megachile rotundata* females were nesting. Treatment sprays occurred on the evening of days 7 (July 5) and 14 (July 12) when bees were not active. The cage experiment was conducted at in North Logan, Utah in 2012.

Treatment Week 1, Week 2, Week 3	Cage	Application regime (date of spray)			Application rate (kg/ha)	
		Week 1 (Days 0–7)	Week 2 (Days 8–14)	Week 3 (Days 15–21)	Week 2	Week 3
NS*, Water**, Water	1	NS	Water	Water	—	—
NS, N-90, N-90	2	NS	N-90	N-90	0.4	0.4
NS, Pristine, Pristine	3	NS	Pristine	Pristine	1.6	1.6
NS, Pristine + N-90, Pristine + N-90	4	NS	Pristine + N-90	Pristine + N-90	2.0	2.0

* NS = no spray; nest initiation period to document normal nesting behavior

** Water was sprayed at equal volumes to treatment sprays

### Experimental Design: *Osmia lignaria* in California

In 2011, six screened field cages (length by width by height = 6.2 × 6.2 × 2 m^3^) were erected over blooming *Phacelia tanacetifolia* Benth. (Hydrophyllaceae) and *Sinapis alba* L. (Brassicaceae) at The Wonderful Company (until 2015 was Paramount Farming Company) in Lost Hills, California. A corrugated plastic nesting box (length by width by height = 22 × 17 × 26 cm^3^) was securely attached to a metal pole 1.5 m above the ground in the center of each cage. The provided artificial cavities were holes drilled into wood blocks each with an accompanying paper straw insert (length by diameter = 15 cm × 7.5 mm). A total of four wood blocks were placed in each nesting box (16 cavities per wood block; 64 cavities per cage; at least 3 cavities per female). Within each cage, a hole was dug and kept wet to provide a mud source for nesting females. Utah-collected *O*. *lignaria* males and females were incubated at The Wonderful Company at 22°C until emergence. Approximately 40 *O*. *lignaria* male and 20 uniquely paint-marked female bees were released in each cage. Activity of individually-marked females was recorded using camcorders for 1 hr and direct observation for 1 hr each day (2 hrs of data collection for each cage per day) between the hours of 0900–1500 PST. To document normal bee behavior before treatments, daily observations were made during Week 1. Nesting behavior data were not recorded on Day 0 (day of bee release in cages) and Day 1, as the females were inspecting multiple cavities as normal nest selection behavior prior to establishing residency in a particular cavity. On the first day of Week 2, treatment sprays occurred at night when bees were not active, and observations were made daily for 1 week. The second sprays similarly were applied for Week 3 and observations were made for 1 week. Treatment and control sprays (Table 1) occurred on the evenings of 14 April (day 7) and 21 April (day 14).

For each *O*. *lignaria* female, mud- and pollen-collecting duration times on observation days were pooled within weeks (Week 1: Days 2–7; Week 2: Days 8–14; Week 3: Days 15–21). That is, we recorded the amount of time taken by individually marked females to leave their nest and return to their nest with either mud (“mud-collecting trip time”) or pollen (“pollen-collecting trip time”). Each day at dusk when females were finished initiating new nest cells, we extracted the paper straw inserts and marked nest progression (i.e., the addition of new cells) by using an indelible marker. Using the nest site observations, we assessed whether females had difficulty recognizing their own nests by recording the number of inspections to her own nest, other nests, or if she entered her own nest without hesitating upon returning from a foraging trip. A score of 1 was given for every time a female began to enter a cavity but then retracted, and a score of zero was given if she directly entered a cavity upon returning from a foraging trip. Nest recognition attempts previously have been used to document confusion at the nesting site due to manipulation of nest recognition cues [69,70].

Our goal for this study was to determine if there were any deaths or behavioral differences after fungicide or adjuvant sprays. We used a repeated measures one-way analysis of variance (ANOVA) for each cage, with the average value for each individually-marked female as the variable repeated each week. Because the cages (i.e., treatment applications and control) were not replicated, we did not perform statistical analyses between treatments. We acknowledge that the replication of the treatments (cages) would be ideal and statistically more rigorous, but we were limited in the number of cages and the human resources for collecting the daily data. Mud- and pollen-collecting trip duration (i.e., time when the female left her nest until the time she returned) and cells produced for each individual female per week were averaged within weeks. Mud-collecting trip time, pollen-collecting trip time, and cell production rate data were log-transformed to satisfy the assumption of normality and homoscedasticity. The repeated measures one-way ANOVA was followed by a standard Bonferroni correction for pairwise comparisons. For the nest recognition attempts data, we also performed repeated measures one-way ANOVAs and Bonferroni corrections for each treatment to test for differences among the number of attempts by returning *O*. *lignaria* females to recognize and enter their own nests or inspect other nests. Attempts to enter nest holes were also averaged for all bees observed within weeks. Significance level was set at 0.05. All data analyses were performed using SPSS version 18.0 (SPSS Inc., Chicago, Illinois).

### Experimental Design: *Megachile rotundata* in Utah

Similar to the *O*. *lignaria* cage study in California, we established four screened field cages (6.2 × 6.2 × 2 m^3^) on planted fields of *Phacelia tanacetifolia* in North Logan, Utah in 2012. A small plywood box (35 × 32 × 28 cm^3^) was affixed to a pole 1.5 m above the ground in the center of each cage to serve as a shelter that held a polystyrene bee board (7 × 7 × 9.5 cm^3^) with 49 cavities. Paper straws were inserted into all cavities, providing two cavities for each *M*. *rotundata* female introduced into the cage. Overwintered prepupae cocooned in leaf-wrapped cells were incubated at 29°C in June to complete bee development to adulthood, and emerged adults were released in cages approximately three weeks later. For each cage, we released 40 males and 20 individually paint-marked females. Activity of individually-marked females was recorded using camcorders for 1 hr and direct observation for 1 hr each day (2 hrs of data collection for each cage per day) between the hours of 0900–1500 MST. To document normal bee behavior before treatments, daily observations were made during Week 1. Nesting behavior data were not recorded on Day 0 (day of bee release in cages) and Day 1 to account for days when bees were only inspecting potential nest sites. On the first day of Week 2, treatment sprays occurred at night when bees were not active, and observations were made daily for 1 week. The second sprays similarly were applied for Week 3, and observations were made for 1 week. Treatment and control sprays occurred on the evenings of 5 July (Day 7) and 12 July (Day 14) (Table 2).

We analyzed our data the same way as for the *O*. *lignaria* cage study, using SPSS version 18.0 (SPSS Inc., Chicago, Illinois). In this *M*. *rotundata* experiment, there were four treatments taking into account the fungicides and adjuvant used. For each week, the mean leaf- and pollen-collecting trip time and cell production rate for each individual female were averaged within weeks (Week 1: Days 2–7; Week 2: Days 8–14; Week 3: Days 15–21), and data were log-transformed to satisfy the assumption of normality and homoscedasticity. The transformed mean leaf- and pollen-collecting trip times and cell production rate per week were compared within treatments using a repeated measures one-way ANOVA followed by a standard Bonferroni correction for pairwise comparisons. For the nest recognition data, attempts to enter nest holes also were averaged for all bees observed within weeks; thus, we performed separate repeated measures one-way ANOVAs and Bonferroni corrections.

### Fungicide Effects on Antennal Response to Odors

Adult *O*. *lignaria* females in cocoons were removed from cold storage and placed in containers in an incubator (25°C) to stimulate emergence. Emerged bees were removed daily and placed into small holding cups and allowed to imbibe syrup that was Pristine-treated or untreated 25% sugar water (25 g sugar/100 ml water) from Day 0 (day of emergence) until they were tested on Day 3. Up to five females were kept in each small container supplied with an unlimited amount of syrup. Only one treatment was applied in which a full field rate of Pristine was suspended in 25% sugar water. For each female on Day 3, an antenna was removed from a chilled live bee, and she was returned to the container. The antenna was affixed across to two electrodes using electrode gel; the electrodes were connected to a signal acquisition controller (IDAC-2, Syntech, Kirchzarten, Germany). Humidified air was blown constantly over the antenna, which was enclosed in a glass tube. A small hole in the side of the glass tube allowed a bee-detectable odor cue to be dispensed from a disposable glass pipette into the air stream that passed over the antenna (Thermo Fisher Scientific, Inc., Waltham, Massachusetts). The cues were puffed in sequence into the air stream at 1 min intervals repeated five times using a stimulus controller (Stimulus Controller CS-55, Syntech, Kirchzarten, Germany). Phenylacetaldehyde and geraniol (Sigma Aldrich Co., St. Louis, Missouri) odors in air served as cues, and air without an odor was presented as a blank. One μl aliquot of an odor was dripped onto a piece of Whatman No. 1 filter paper (2 cm^2^) (Sigma Aldrich Co., St. Louis, Missouri). Using forceps, the filter paper was inserted into the glass pipette up to the tapered portion before the tip and crimped so that air was allowed to pass across the paper and exit the pipette into the glass tube for exposure to the antenna. After completion of the test on the first antenna, the second antenna was excised from the same bee and tested; the bee was then freezer-killed. For each bee, each antenna was treated as a separate right or left sample.

Antennal responses (amplitudes) were measured with the Syntech EAG Software (Kirchzarten, Germany), and were compared for the effects of treatment (fungicide exposed or not) and odor cue and the repeated effect of the delivery sequence (when stimulus was delivered from first to fifth series in the sequence) for each within test replicate using PROC MIXED in SAS version 9.2 (SAS Institute Inc., North Carolina). Random effects included in the model were sample and whether the antenna was from the left or right position of the head.

### Ethics Statement

Dr. Gordon Wardell of The Wonderful Company gave permission to conduct the study for the California site and Dr. Raymond Cartee (now retired), Research Farms Director of Utah State University, gave permission to conduct the study for the Utah site.

## Results

### Cage Study: *Osmia lignaria* in California

**Fig 1:**
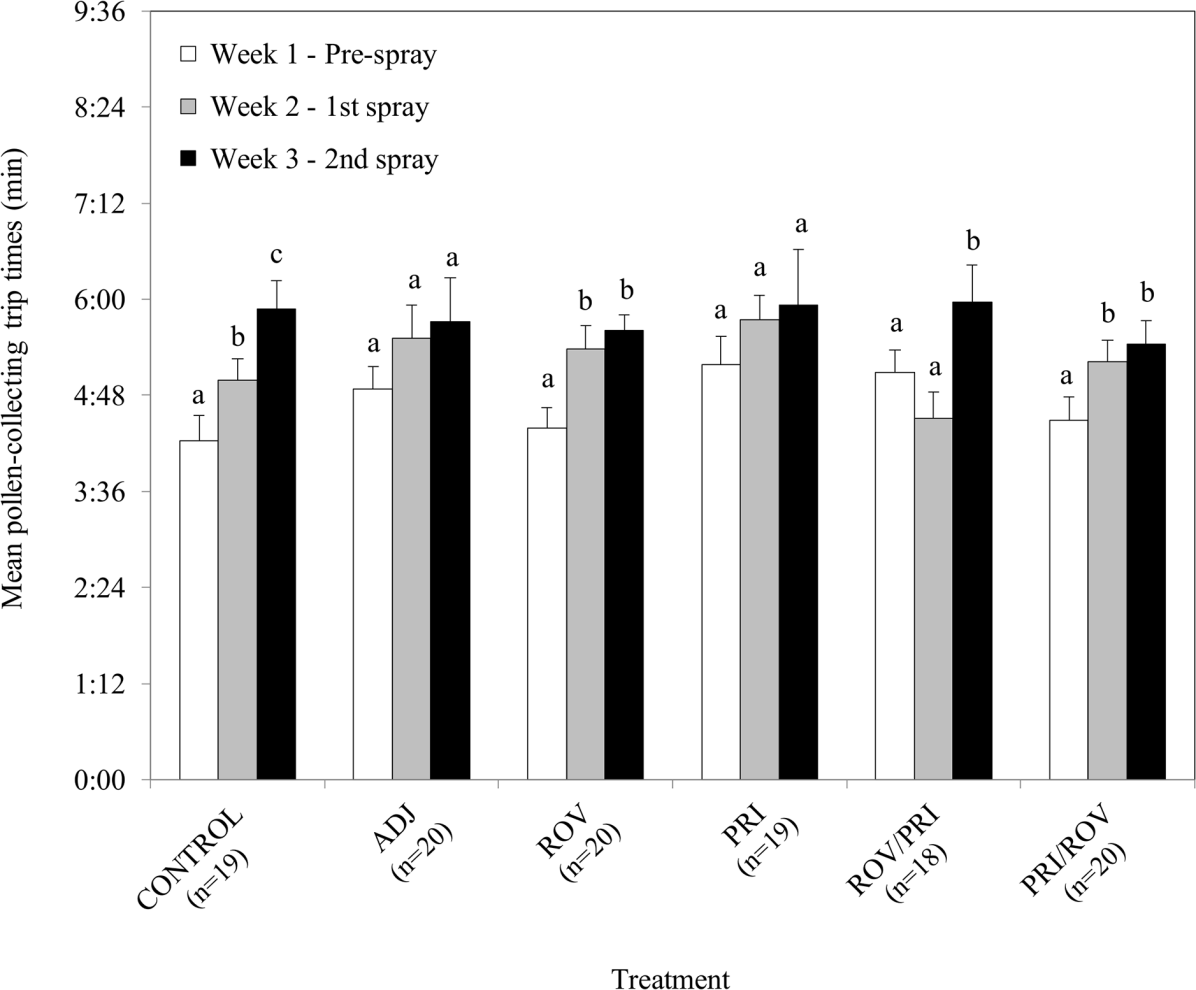
Mean pollen-collecting trip times by *Osmia lignaria* females before and after treatment applications. Means within each treatment with different letters are significantly different (*P* < 0.05).

Mean pollen-collecting trip times increased significantly between weeks by *O*. *lignaria* females for the CONTROL, ROV, ROV/PRI, and PRI/ROV treatments (Fig 1, Table 3). No significant differences were detected between weeks for ADJ or PRI treatments (Fig 1, Table 3). Pairwise comparisons indicated that *O*. *lignaria* females took longer to collect pollen in the control cage in Week 2 than in Week 1 (*t*_*6*_ = 8.82, *P* < 0.0001) and longer in Week 3 than in Week 2 (*t*_*6*_ = 7.24, *P* < 0.0001). Pollen-collecting trip times significantly increased from Week 1 to Week 2 for ROV (*t*_*6*_ = 5.55, *P* < 0.001) and PRI/ROV (*t*_*6*_ = 4.67, *P* = 0.024). Pollen-collecting trip times significantly increased from Week 2 to Week 3 for ROV/PRI (*t*_*6*_ = 8.25, *P* < 0.0001). No significant differences on mean mud-collecting trip times were found for any of the treatments and control (pooled Week 1 range: 1:22–2:11 min; pooled Week 2 range: 1:04–4:01 min; pooled Week 3 range: 1:04–3:33 min) (Table 3). Cell production per day per *O*. *lignaria* female was less in the third week than in the first week of the study in all cages, but these differences were not significant (S1 Fig). There were, however, slightly lower mean cell production rates after the sprays in the treatment cages (except for the ROV/PRI treatment) compared to the control, and greater reduction of the number of cells produced over time within treatment cages, perhaps suggesting a treatment effect on productivity (Table 3, S1 Table).

**Table 3:** ANOVA results for the treatment effects on the pollen-collecting trip times, mud-collecting trip times, cell production rates per day, and the number of nest recognition attempts made to enter her own and other nests by *Osmia lignaria* females in cages in Lost Hills, California in 2011.

	Treatment					
Dependent Variable	CONTROL	ADJ	ROV	PRI	ROV/PRI	PRI/ROV
Pollen-collecting trip time	*F*_2,136_ = 8.48, ***P* < 0.001**	*F*_2,155_ = 0.98, *P* = 0.451	*F*_2,145_ = 6.54, ***P =* 0.002**	*F*_2,128_ = 0.68, *P =* 0.412	*F*_2,114_ = 5.42, ***P* < 0.001**	*F*_2,139_ = 3.34, ***P =* 0.035**
Mud-collecting trip time	*F*_2,139_ = 0.69, *P* = 0.412	*F*_2,201_ = 1.12, *P* = 0.331	*F*_2,111_ = 0.77, *P* = 0.387	*F*_2,162_ = 0.28, *P* = 0.834	*F*_2,127_ = 0.30, *P* = 0.744	*F*_2,172_ = 0.98, *P* = 0.314
Cell production rate per day	*F*_2,84_ = 0.11, *P* = 0.922	*F*_2,67_ = 1.55, *P* = 0.181	*F*_2,58_ = 1.18, *P* = 0.274	*F*_2,87_ = 1.60, *P* = 0.144	*F*_2,93_ = 0.97, *P* = 0.352	*F*_2,76_ = 1.48, *P* = 0.229
Nest recognition attempts—Own Nest	*F*_2,261_ = 10.87, ***P* < 0.0001**	*F*_2,240_ = 9.47, ***P* < 0.0001**	*F*_2,311_ = 7.68, ***P* < 0.0001**	*F*_2,278_ = 12.24, ***P* < 0.0001**	*F*_2,354_ = 9.97, ***P* < 0.0001**	*F*_2,401_ = 11.95, ***P* < 0.0001**
Nest recognition attempts—Other Nests	*F*_2,301_ = 8.11, ***P =* 0.004**	*F*_2,288_ = 10.24, ***P* < 0.0001**	*F*_2,197_ = 8.01, ***P =* 0.007**	*F*_2,412_ = 17.40, ***P* < 0.0001**	*F*_2,341_ = 13.24, ***P* < 0.0001**	*F*_2,377_ = 10.88, ***P* < 0.0001**

**Fig 2:**
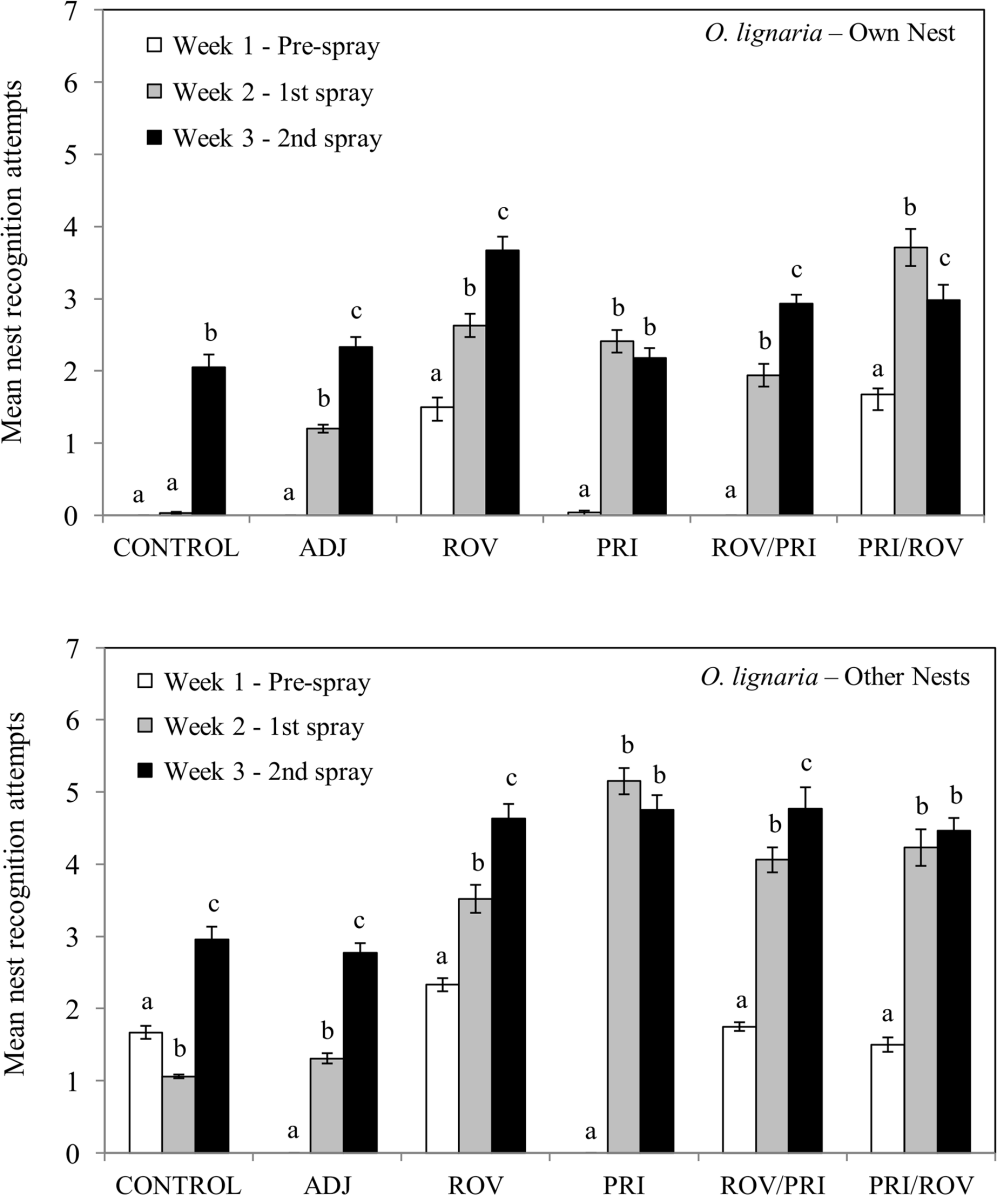
Mean attempts to enter her nest and other nests by *Osmia lignaria* females before and after treatment applications. Means within each treatment with different letters are significantly different (*P* < 0.05).

The mean attempts by *O*. *lignaria* females to enter their own nest cavity and other nest cavities were significantly different between weeks in all treatments and in the control (Fig 2, Table 3). Post hoc tests indicated that there were no significant increases in nest attempts between Weeks 1 and 2 for CONTROL, but mean attempts by females to enter her own nest or other nests increased in Week 3 (Fig 2, S2 Table). For all treatments, attempts at their own or other nest holes increased between Week 1 and Week 2. For ADJ, ROV, ROV/PRI, there also were significantly more attempts to enter their own and other nests in Week 3 compared to Week 2 (Fig 2, S2 and S3 Tables). Attempts to enter their own nest decreased slightly between Weeks 2 and 3 for PRI and PRI/ROV treatments; attempts to enter other nests also decreased for the PRI treatment for these weeks (Fig 2, S3 Table). The increase in the total number of nest entry attempts in treatment cages clearly shows overall differences (Table 3).

### Cage Study: *Megachile rotundata* in Utah

**Fig 3:**
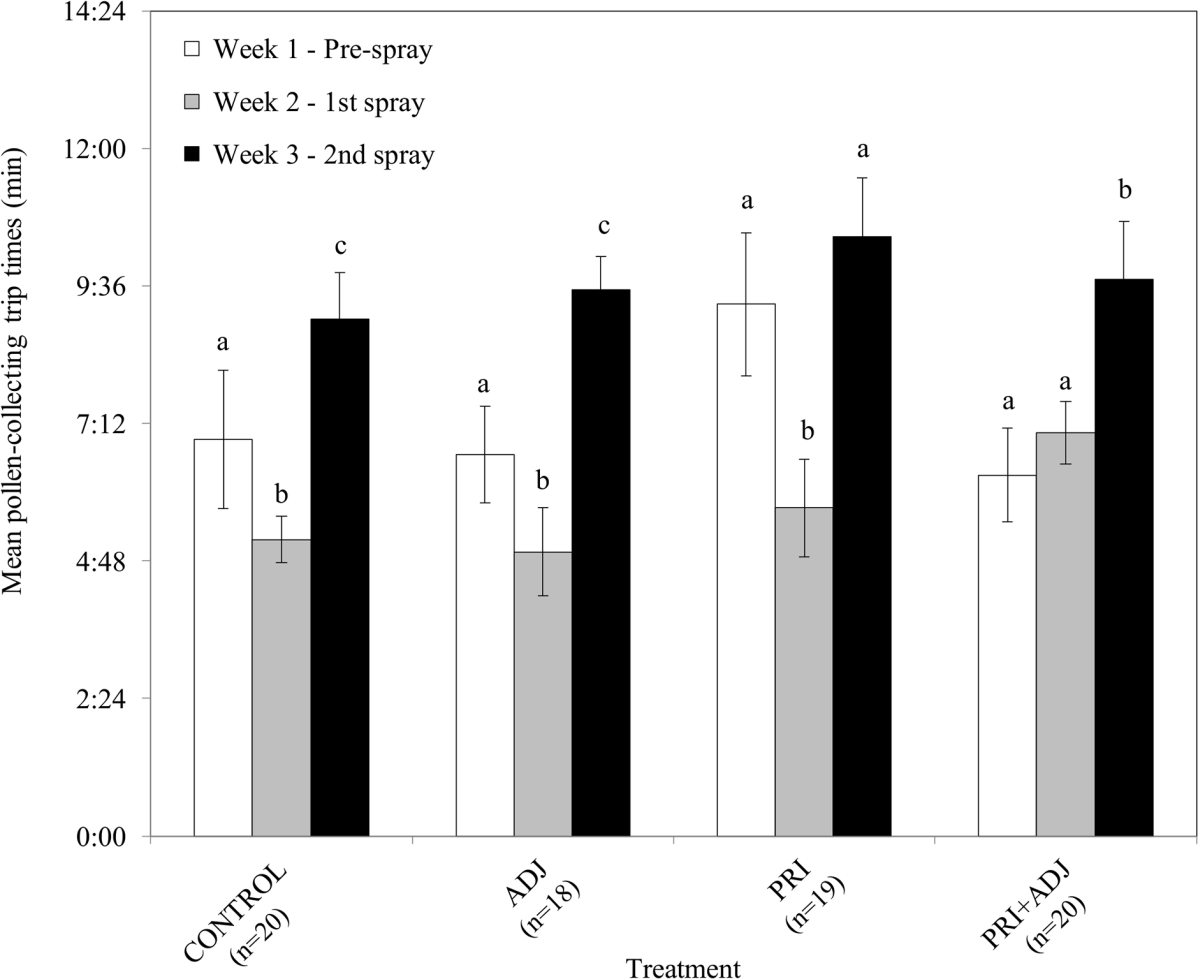
Mean pollen-collecting trip times by *Megachile rotundata* females before and after treatment applications. Means within each treatment with different letters are significantly different (*P* < 0.05).

**Fig 4:**
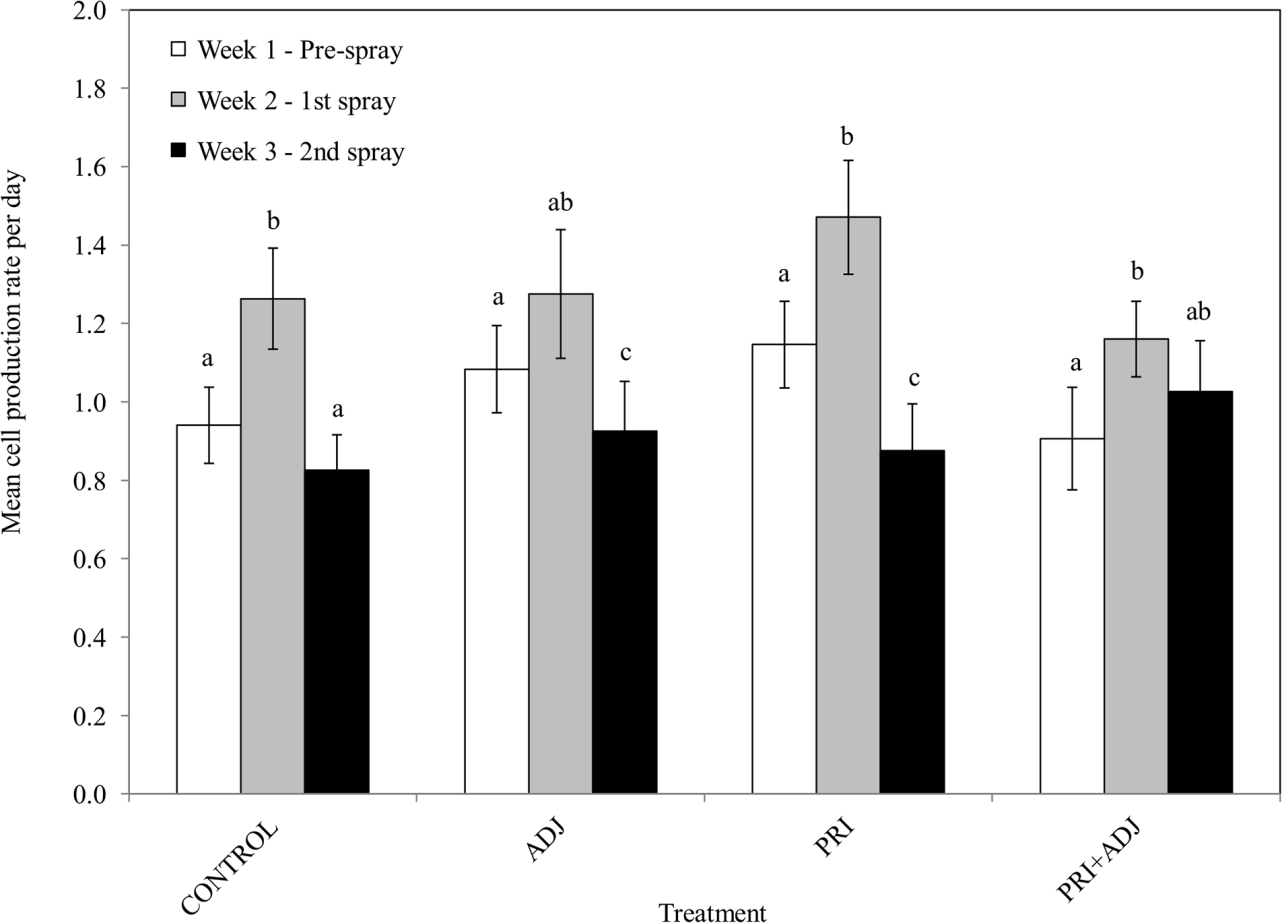
Mean cell production rate per day by *Megachile rotundata* females before and after treatment applications. Means within each treatment with different letters are significantly different (*P* < 0.05).

For *M*. *rotundata* females, significant differences in mean pollen-collecting trip times between weeks were revealed for all treatments and control (Fig 3, Table 4). *M*. *rotundata* females took longer to collect pollen in CONTROL, ADJ, and PRI+ADJ in Week 3 compared to Weeks 1 and 2 (Fig 3, Table 4). Females in the CONTROL cage took longer to collect pollen in Week 1 than in Week 2 (*t*_*6*_ = 9.77, *P* < 0.0001), and in Week 3 than in Week 2 (*t*_*6*_ = 6.47, *P* < 0.0001). In the ADJ cage, pollen-collecting times increased significantly in Week 3 compared to Week 1 (*t*_*6*_ = 8.78, *P* < 0.0001) and Week 2 (*t*_*6*_ = 10.33, *P* < 0.0001). Pollen-collecting trip times in the PRI treatment significantly decreased in Week 2 from Week 1 (*t*_*6*_ = 6.58, *P* < 0.001) and then significantly increased in Week 3 (*t*_*6*_ = 8.23, *P* < 0.001). Similarly, mean pollen-collecting trip times were significantly longer in Week 3 in the PRI+ADJ treatment than in Week 1 (*t*_*6*_ = 6.89, *P <* 0.001) and Week 2 (*t*_*6*_ = 7.23, *P* = 0.033). No significant differences were obvious for the relative increases in mean leaf-collecting trip times for any of the treatments and control, with much variation in the times recorded (pooled Week 1 range: 0:36–6:52 min; pooled Week 2 range: 0:21–13:18 min; pooled Week 3 range: 0:48–10:21 min; Table 4). Mean cell production rate per day by *M*. *rotundata* females was significantly higher in Week 2 compared to Weeks 1 and 3 for all treatments and control except for the ADJ and PRI + ADJ cages (Fig 4, Table 4, S4 Table).

**Table 4:** ANOVA results for the treatment effects on the pollen-collecting trip time, leaf-collecting trip time, cell production rates per day, and the number of nest recognition attempts made to enter her own and other nests by *Megachile rotundata* females in cages in North Logan, Utah in 2012.

	Treatment			
Dependent Variable	CONTROL	ADJ	PRI	PRI+AJ
Pollen-collecting trip time	*F*_2,241_ = 9.24, ***P* < 0.0001**	*F*_2,202_ = 7.78, ***P* < 0.0001**	*F*_2,145_ = 5.98, ***P* < 0.0001**	*F*_2,128_ = 6.61, ***P* < 0.0001**
Leaf-collecting trip time	*F*_2,157_ = 1.02, *P* = 0.425	*F*_2,201_ = 0.88, *P* = 0.523	*F*_2,257_ = 0.72, *P* = 0.687	*F*_2,314_ = 1.10, *P* = 0.384
Cell production rate per day	*F*_2,222_ = 4.30, ***P* = 0.006**	*F*_2,287_ = 5.12, ***P* = 0.004**	*F*_2,197_ = 8.31, ***P* < 0.0001**	*F*_2,175_ = 4.62, ***P* = 0.002**
Nest recognition attempts—Own Nest	*F*_2,261_ = 6.87, ***P* < 0.0001**	*F*_2,240_ = 7.72, ***P* < 0.0001**	*F*_2,311_ = 11.33, ***P* < 0.0001**	*F*_2,278_ = 15.55, ***P* < 0.0001**
Nest recognition attempts—Other Nests	*F*_2,240_ = 7.51, ***P* < 0.0001**	*F*_2,187_ = 5.98, ***P* = 0.005**	*F*_2,320_ = 12.34, ***P* < 0.0001**	*F*_2,345_ = 10.58, ***P* < 0.0001**

**Fig 5:**
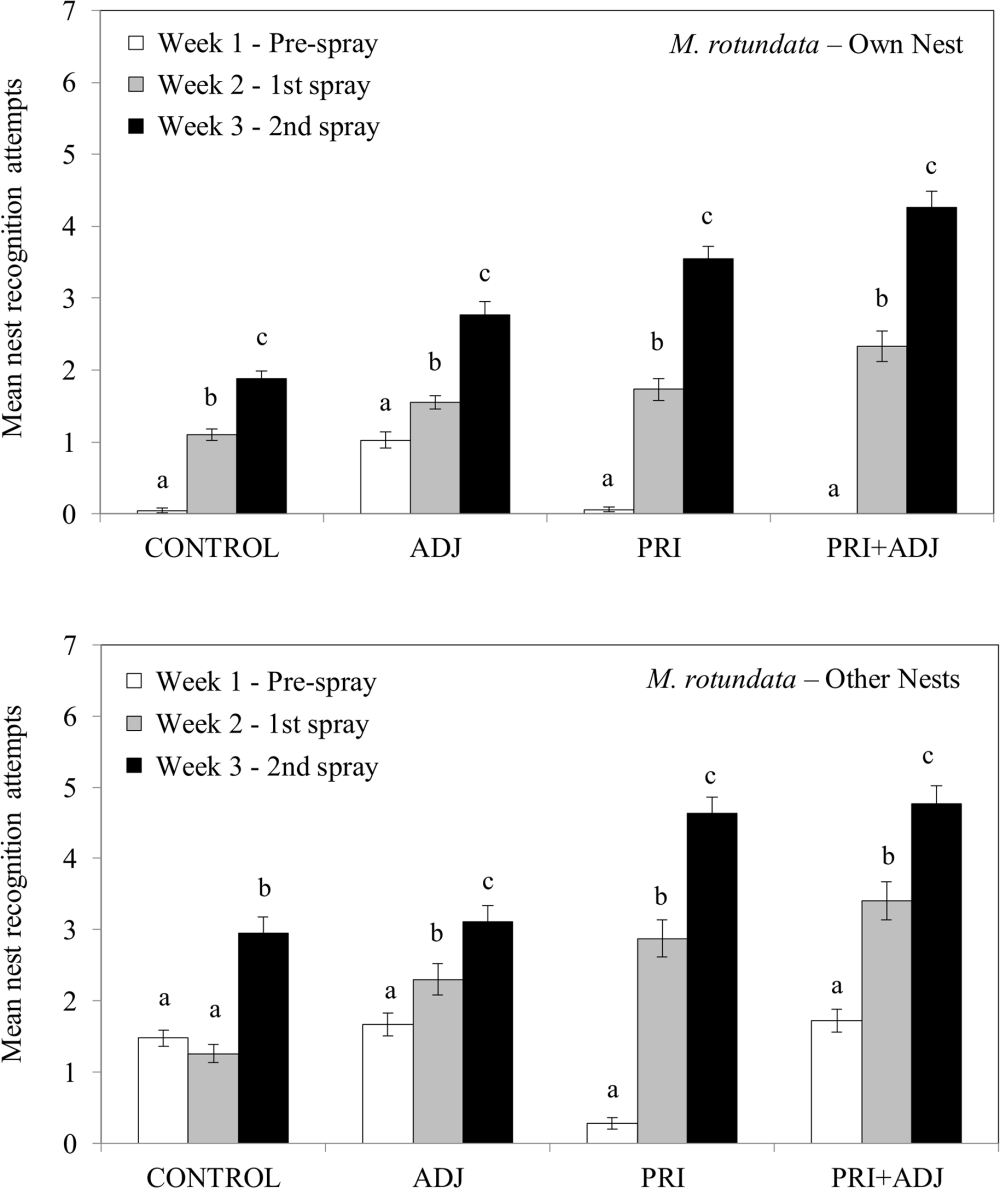
Mean attempts to enter her nest and other nests by *Megachile rotundata* females before and after treatment applications. Means within each treatment with different letters are significantly different (*P* < 0.05).

Significant differences in mean attempts by *M*. *rotundata* females to enter their own nest cavity and other nest cavities were found for all treatments and the control (Fig 5, Table 4). Except for mean attempts to enter other holes in the CONTROL cage, attempts by females to enter their own and other nest cavities increased significantly each week in all cages (Fig 5, S5 and S6 Tables). Females nesting in the CONTROL cage had noticeably fewer mean number of attempts in Weeks 2 and 3 than did females in ADJ, PRI, and PRI+ADJ cages (Fig 5, S5 and S6 Tables). The increase in the total number of nest entry attempts in treatment cages clearly shows overall differences (Fig 5, Table 4).

### Fungicide Effects on Antennal Response to Odors

There was a significant difference between EAG response to cues (*F*_1,40.9_ = 66.39, *P* < 0.001) and occurrence of the cue (*F*_1,254_ = 6.46, *P* < 0.001). However, there was no effect of the ingestion of fungicide syrup on response to cues compared to the control (*F*_1,22.9_ = 0.11, *P* = 0.7465) and no interaction effects of any of the variables. The presence of the fungicide in the sugar water did not inhibit the bees’ ability to discriminate between floral cues. Also, the waning of the intensity of the response throughout the sequential delivery of the cues was not affected by the ingestion of the fungicide.

## Discussion

Studies of both social and solitary bees are imperative for assessing the incidence, exposure routes, and risk of harmful effects of pesticides [71]. Sublethal effects that disrupt foraging and nesting by solitary bees can lead directly and immediately to a reduction in progeny number and survival to the next generation, compared to the reproductive success in social bees that may be able to recover from a loss of replaceable workers. Our limited, semi-field cage study with *O*. *lignaria* staged scenarios that mimicked the back to back fungicide sprays applied during almond bloom in California orchards. For comparison, we also applied similar scenarios to another commercial solitary bee, *M*. *rotundata*, to assure that the effects were common to other cavity-nesting bees. Indeed, we observed and documented reduction in nest recognition ability in both of these two important managed bees, providing the first empirical evidence of the sublethal, but not lethal, effects of fungicide and adjuvant sprays. *O*. *lignaria* females increased (2–7 times) their number of attempts to enter their own nest cavities as well as other cavities after being exposed to the fungicides Rovral 4F and Pristine and the adjuvant N-90. Similarly, more attempts (2–7 times) by *M*. *rotundata* females to enter their own nest cavities as well as checking other nest cavities were observed after sprays of N-90 adjuvant, Pristine only, and Pristine + N-90 adjuvant. The evidence of disorientation at the nest site in a cage setting implies some loss of memory or sensory ability that may have detrimental impacts on retention of bees at nest sites in commercial orchards.

Other possible sublethal effects of the fungicides and adjuvant on foraging times and cell production were not detected. For *O*. *lignaria*, foraging times increased each week, except for in the ROV/PRI cage. For *M*. *rotundata*, foraging times decreased in Week 2 and then increased in Week 3 beyond Week 1 levels. However, the change in foraging times appears to be an effect of time and not treatment. Also, we did not perform rigorous inspections and examinations of larval development, and, therefore, we cannot confirm or deny conspicuous sublethal effects on larval survival or the timing of larval development (i.e., delay or cessation of growth and development) for either species.

A previous study found that contact and oral exposures to high doses of Rovral were not lethal to *O*. *lignaria* and *A*. *mellifera* adults [36], and accordingly, exposure after sprays with Rovral in our study did not affect *O*. *lignaria* adult survival. Ladurner et al. [48] also tested Rovral in cage studies with *O*. *lignaria* in Utah and found no lethal or behavioral effects of this fungicide. In our study, however, we did detect a reduction in nest recognition ability. One possible reason that we were able to detect this sublethal behavioral effect was that the flowable formulation in our study allowed for a different amount or direct exposure than the wettable powder formulation tested in the Ladurner et al. [48] study. Another plausible explanation was that their study was carried out in Utah in June when the average weekly temperature was 85°F. Our study was conducted in California in April when the average weekly temperature was 60°F, suggesting that perhaps temperature, humidity, or other environmental factors may have influenced the persistence of the chemicals in the cages or affected the activity of these spring bees and their oral and/or contact exposure to the fungicide and adjuvant residues.

Scent marking of nests by gregariously nesting solitary bees, such as *O*. *lignaria* and *M*. *rotundata*, aids in nest location and recognition by females returning to their nests [69–72]. Nesting *O*. *lignaria* and *M*. *rotundata* females likely use a combination of visual cues and landmarks to guide them to the nest site [73], but employ olfactory cues at short ranges to pinpoint their individual nest tunnels [69,70], as has been shown in other bee species [74–76]. Guédot et al. [69] demonstrated that removing parts of nesting tubes, particularly the outer section of nesting tubes that includes the nest entrance, from test *O*. *lignaria* females and replacing them with new (unmarked) nesting tubes caused nest recognition impairment. Females were disoriented, leading to the inspection of many different holes and searching for their own uniquely-marked nest entrance. Furthermore, in experimental tests with *O*. *lignaria* females, replacing sections of the original nests and presenting them to the females again, 10 out of the 15 *O*. *lignaria* females abandoned their nests altogether [69]. Similar work in *M*. *rotundata* also showed the use of a nest recognition cue, and also confusion at the nest site when bee-marked tube sections were removed. The effects of the fungicides and the adjuvant found in our study were similar to this nest recognition inhibition in *O*. *lignaria* and *M*. *rotundata* in the Guédot et al. studies [69,70].

Our EAG analyses from *O*. *lignaria* females suggest that the disorientation of *O*. *lignaria* females at their nests was not due to an inhibition of antennal stimulus detection, such as the detection of a nest recognition cue. With or without oral exposure to fungicides in sugar water, *O*. *lignaria* antennae responded similarly to odor cues, with some odors eliciting significantly greater responses than others. The fungicide did not change the level of response to odor cues due to some physical disturbance or neural disruption of the antennal sensory apparatus. An explanation for the disorientation behavior observed in our study is, therefore, not at the interface of a detectable odor and the receptor site. But it is possible that the fungicides and adjuvant impaired olfactory-receptor-neuron processing by blocking receptors specific to certain nest odors and their neural activity in the bee brain or beyond, thus affecting memory and nest recognition.

Effects of insecticides on bee learning and memory have been documented or suspected in previous studies. Aliouane et al. [77] demonstrated that subchronic exposure to thiamethoxam, a systemic neonicotinoid, led to a decrease of olfactory memory and impairment of *A*. *mellifera* learning. Williams & Wright [78] also demonstrated learning and memory impairment in *A*. *mellifera* after exposure to sublethal doses of certain pesticides. After exposure to imidacloprid, coumaphos (organophosphate acetylcholinesterase inhibitor), and a combination of the two compounds, *A*. *mellifera* foragers showed an inability to differentiate between a conditioned odor from a novel odor during memory tests [78]. The behavioral disruption in nest recognition by *O*. *lignaria* and *M*. *rotundata* females in our study now provides evidence that certain fungicides also may impair memory, learning, foraging, and nest recognition abilities in bees, and in this case in solitary bees.

The nest location impairment after exposure to fungicide sprays revealed in our study supports the anecdotal evidence of abandonment of nests by *O*. *lignaria* after fungicide sprays, particularly in California almond orchards and cherries [48]. Because fungicides are not commonly used in alfalfa fields where *M*. *rotundata* are used as pollinators, no anecdotes of similar nest abandonment behaviours are known for this widely-used species. In our study, if our bees had not been caged, it is possible that they would have abandoned their nests. Open-field investigations are needed to further corroborate findings from our semi-field experiments.

A growing number of empirical studies also document the differences between bee species and life stages in their sensitivity to various pesticides. Ladurner et al. [36] found striking mortality rates for *O*. *lignaria* after oral administration of propiconazole, a broad spectrum fungicide, compare to a contact application that was not lethal. Huntzinger et al. [51] found that high concentrations of Rovral 4F and Rovral 50 WP increased larval mortality when applied directly to mass provisions of *M*. *rotundata*. Recently, Arena & Sgolastra [79] compared the sensitivity to various pesticides between *A*. *mellifera* and non-*Apis* bees, and a high degree of variability in the sensitivity among bee species was found. Biddinger et al. [39] further demonstrated that the decreasing order of toxicity to several types of insecticides differed between *O*. *cornifrons* and *A*. *mellifera*. This study also showed that the orchard fungicide fenbuconazole, when combined with acetamiprid, was five times more toxic to *A*. *mellifera* and two times more toxic to *O*. *lignaria* than the fungicide alone.

Exposure to a presumed benign substance, the N-90 non-ionic spray adjuvant also caused some impairment of nest recognition ability in both *O*. *lignaria* and *M*. *rotundata* females. Ciarlo et al. [80] tested different agricultural spray adjuvants typically used on almonds in California. They found *A*. *mellifera* olfactory learning was impaired after bees ingested different adjuvants, particularly organosilicone adjuvants, but also one non-ionic adjuvant. Our findings that a non-ionic spray adjuvant disrupted solitary bee nest recognition ability highlight the importance of future testing of agricultural additives to safeguard bees in agroecosystems.

Not only does our study provide conclusive evidence of a sublethal effect of commonly-used fungicides on bee behavior, but it also defines a behavior that can be quantified in future studies in semi-field or field trials. Other variables should also be considered, such as larval development, winter survival of progeny, and nesting success of the next generation. Even though we did not see acute toxicity in adult bees or a significant reduction in cell production rate in both bee species, it is imperative to understand the proximate causes of the effects of sublethal doses of fungicides and fungicide-containing tank mixtures that may impair, disrupt, or inhibit different sensory signaling systems in social and solitary bees. Once understood, the properties of the fungicides that contribute to this effect may be identified and possibly removed from the product formulation. Effective crop protection fungicides are necessary, but finding or developing those with formulations that do not adversely affect the required pollinators would help alleviate the problems related to fungicide use.

## Supporting Information

S1 Fig Mean cell production rate per day by *Osmia lignaria* females before and after treatment applications. (DOCX)

S1 Table Bonferroni-corrected post-hoc tests of within-treatment mean cell production rate per day by *Osmia lignaria* females before and after fungicide and adjuvant sprays in a cage study in Lost Hills, California in 2011. (DOCX)

S2 Table Bonferroni-corrected post-hoc tests of within-treatment mean nest recognition attempts by *Osmia lignaria* females to enter her own nest before and after fungicide and adjuvant sprays in a cage study in Lost Hills, California in 2011. (DOCX)

S3 Table Bonferroni-corrected post-hoc tests of within-treatment mean nest recognition attempts by *Osmia lignaria* females to enter other nests before and after fungicide and adjuvant sprays in a cage study in Lost Hills, California in 2011. (DOCX)

S4 Table Bonferroni-corrected post-hoc tests of within-treatment mean cell production rate per day by *Megachile rotundata* females before and after fungicide and adjuvant sprays in a cage study in North Logan, Utah in 2012. (DOCX)

S5 Table Bonferroni-corrected post-hoc tests of within-treatment mean nest recognition attempts by *Megachile rotundata* females to enter her own nest before and after fungicide and adjuvant sprays in a cage study in North Logan, Utah in 2012. (DOCX)

S6 Table Bonferroni-corrected post-hoc tests of within-treatment mean nest recognition attempts by *Megachile rotundata* females to enter her other nests before and after fungicide and adjuvant sprays in a cage study in North Logan, Utah in 2012. (DOCX)
