# Supplementary material for: Effects of Fungicide and Adjuvant Sprays on Nesting Behavior in Two Managed Solitary Bees, Osmia lignaria and Megachile rotundata
Source: PLoS One. 2015 Aug 14;10(8):e0135688. doi: 10.1371/journal.pone.0135688 (PMC4537283; doi:10.1371/journal.pone.0135688)
Supplement: S1 Fig — (DOCX) [file pone.0135688.s001.docx]

**Figure S1.** Mean cell production rate per day by *Osmia lignaria* females before and after treatment applications in Lost Hills, California in 2011.
