# Supplementary material for: Effects of Fungicide and Adjuvant Sprays on Nesting Behavior in Two Managed Solitary Bees, Osmia lignaria and Megachile rotundata
Source: PLoS One. 2015 Aug 14;10(8):e0135688. doi: 10.1371/journal.pone.0135688 (PMC4537283; doi:10.1371/journal.pone.0135688)
Supplement: S1 Table — (DOCX) [file pone.0135688.s002.docx]

**Table S1*.*** Bonferroni-corrected post-hoc tests of within-treatment mean cell production rate per day by *Osmia lignaria* females before and after fungicide and adjuvant sprays in a cage study in Lost Hills, California in 2011.

| Effect | SE | *t* | Adj *P* |
| --- | --- | --- | --- |
| Control – Week 1 × Week 2 | 0.428 | 0.52 | 0.875 |
| Control – Week 1 × Week 3 | 0.382 | 0.33 | 0.921 |
| Control – Week 2 × Week 3 | 0.433 | 0.36 | 0.908 |
| ADJ – Week 1 × Week 2 | 0.327 | 0.72 | 0.587 |
| ADJ – Week 1 × Week 3 | 0.439 | 2.02 | 0.092 |
| ADJ – Week 2 × Week 3 | 0.299 | 1.84 | 0.257 |
| ROV – Week 1 × Week 2 | 0.287 | 0.98 | 0.421 |
| ROV – Week 1 × Week 3 | 0.252 | 1.04 | 0.487 |
| ROV – Week 2 × Week 3 | 0.301 | 0.29 | 0.940 |
| PRI – Week 1 × Week 2 | 0.249 | 2.45 | 0.071 |
| PRI – Week 1 × Week 3 | 0.354 | 1.21 | 0.545 |
| PRI – Week 2 × Week 3 | 0.423 | 0.65 | 0.845 |
| ROV/PRI – Week 1 × Week 2 | 0.297 | 1.23 | 0.657 |
| ROV/PRI – Week 1 × Week 3 | 0.345 | 1.02 | 0.846 |
| ROV/PRI – Week 2 × Week 3 | 0.275 | 0.54 | 0.899 |
| PRI/ROV – Week 1 × Week 2 | 0.215 | 1.10 | 0.621 |
| PRI/ROV – Week 1 × Week 3 | 0.234 | 2.31 | 0.214 |
| PRI/ROV – Week 2 × Week 3 | 0.197 | 1.54 | 0.771 |
