# Supplementary material for: Effects of Fungicide and Adjuvant Sprays on Nesting Behavior in Two Managed Solitary Bees, Osmia lignaria and Megachile rotundata
Source: PLoS One. 2015 Aug 14;10(8):e0135688. doi: 10.1371/journal.pone.0135688 (PMC4537283; doi:10.1371/journal.pone.0135688)
Supplement: S2 Table — (DOCX) [file pone.0135688.s003.docx]

**Table S2*.*** Bonferroni-corrected post-hoc tests of within-treatment mean nest recognition attempts by *Osmia lignaria* females to enter her own nest before and after fungicide and adjuvant sprays in a cage study in Lost Hills, California in 2011.

| Effect | SE | *t* | Adj *P* |
| --- | --- | --- | --- |
| Control – Week 1 × Week 3 | 0.076 | 27.01 | <0.0001 |
| Control – Week 2 × Week 3 | 0.083 | 24.41 | <0.0001 |
| ADJ – Week 1 × Week 2 | 0.122 | 9.87 | <0.0001 |
| ADJ – Week 1 × Week 3 | 0.104 | 22.37 | <0.0001 |
| ADJ – Week 2 × Week 3 | 0.130 | 8.63 | <0.0001 |
| ROV – Week 1 × Week 2 | 0.307 | 3.69 | 0.001 |
| ROV – Week 1 × Week 3 | 0.329 | 6.60 | <0.0001 |
| ROV – Week 2 × Week 3 | 0.238 | 4.37 | <0.0001 |
| PRI – Week 1 × Week 2 | 0.158 | 14.99 | <0.0001 |
| PRI – Week 1 × Week 3 | 0.167 | 12.77 | <0.0001 |
| ROV/PRI – Week 1 × Week 2 | 0.141 | 13.77 | <0.0001 |
| ROV/PRI – Week 1 × Week 3 | 0.121 | 24.20 | <0.0001 |
| ROV/PRI – Week 2 × Week 3 | 0.149 | 6.62 | <0.0001 |
| PRI/ROV – Week 1 × Week 2 | 0.216 | 9.43 | <0.0001 |
| PRI/ROV – Week 1 × Week 3 | 0.248 | 5.24 | <0.0001 |
| PRI/ROV – Week 2 × Week 3 | 0.280 | 2.63 | 0.027 |
