# Supplementary material for: Effects of Fungicide and Adjuvant Sprays on Nesting Behavior in Two Managed Solitary Bees, Osmia lignaria and Megachile rotundata
Source: PLoS One. 2015 Aug 14;10(8):e0135688. doi: 10.1371/journal.pone.0135688 (PMC4537283; doi:10.1371/journal.pone.0135688)
Supplement: S3 Table — (DOCX) [file pone.0135688.s004.docx]

**Table S3*.*** Bonferroni-corrected post-hoc tests of within-treatment mean nest recognition attempts by *Osmia lignaria* females to enter other nests before and after fungicide and adjuvant sprays in a cage study in Lost Hills, California in 2011.

| Effect | SE | *t* | Adj *P* |
| --- | --- | --- | --- |
| Control – Week 1 × Week 2 | 0.174 | 3.49 | 0.002 |
| Control – Week 1 × Week 2 | 0.178 | 7.26 | <0.0001 |
| Control – Week 2 × Week 3 | 0.154 | 12.34 | <0.0001 |
| ADJ – Week 1 × Week 2 | 0.194 | 6.77 | <0.0001 |
| ADJ – Week 1 × Week 3 | 0.162 | 17.13 | <0.0001 |
| ADJ – Week 2 × Week 3 | 0.175 | 8.35 | <0.0001 |
| ROV – Week 1 × Week 2 | 0.221 | 5.37 | <0.0001 |
| ROV – Week 1 × Week 3 | 0.226 | 10.18 | <0.0001 |
| ROV – Week 2 × Week 3 | 0.237 | 4.70 | <0.0001 |
| PRI – Week 1 × Week 2 | 0.261 | 19.75 | <0.0001 |
| PRI – Week 1 × Week 3 | 0.258 | 18.42 | <0.0001 |
| ROV/PRI – Week 1 × Week 2 | 0.228 | 10.15 | <0.0001 |
| ROV/PRI – Week 1 × Week 3 | 0.266 | 11.35 | <0.0001 |
| ROV/PRI – Week 2 × Week 3 | 0.261 | 2.70 | 0.022 |
| PRI/ROV – Week 1 × Week 2 | 0.387 | 7.05 | <0.0001 |
| PRI/ROV – Week 1 × Week 3 | 0.321 | 9.21 | <0.0001 |
