# Supplementary material for: Effects of Fungicide and Adjuvant Sprays on Nesting Behavior in Two Managed Solitary Bees, Osmia lignaria and Megachile rotundata
Source: PLoS One. 2015 Aug 14;10(8):e0135688. doi: 10.1371/journal.pone.0135688 (PMC4537283; doi:10.1371/journal.pone.0135688)
Supplement: S4 Table — (DOCX) [file pone.0135688.s005.docx]

**Table S4*.*** Bonferroni-corrected post-hoc tests of within-treatment mean cell production rate per day by *Megachile rotundata* females before and after fungicide and adjuvant sprays in a cage study in North Logan, Utah in 2012.

| Effect | SE | *t* | Adj *P* |
| --- | --- | --- | --- |
| Control – Week 1 × Week 2 | 0.256 | 6.42 | <0.0001 |
| Control – Week 1 × Week 3 | 0.212 | 0.55 | 0.756 |
| Control – Week 2 × Week 3 | 0.341 | 8.62 | <0.0001 |
| ADJ – Week 1 × Week 2 | 0.288 | 2.01 | 0.112 |
| ADJ – Week 1 × Week 3 | 0.247 | 4.22 | 0.035 |
| ADJ – Week 2 × Week 3 | 0.324 | 5.98 | <0.0001 |
| PRI – Week 1 × Week 2 | 0.255 | 3.87 | 0.002 |
| PRI – Week 1 × Week 3 | 0.301 | 5.32 | <0.0001 |
| PRI – Week 2 × Week 3 | 0.247 | 10.54 | <0.0001 |
| PRI+ADJ – Week 1 × Week 2 | 0.273 | 4.31 | 0.024 |
| PRI+ADJ – Week 1 × Week 3 | 0.211 | 1.33 | 0.754 |
| PRI+ADJ – Week 2 × Week 3 | 0.321 | 0.95 | 0.826 |
