# Supplementary material for: Effects of Fungicide and Adjuvant Sprays on Nesting Behavior in Two Managed Solitary Bees, Osmia lignaria and Megachile rotundata
Source: PLoS One. 2015 Aug 14;10(8):e0135688. doi: 10.1371/journal.pone.0135688 (PMC4537283; doi:10.1371/journal.pone.0135688)
Supplement: S5 Table — (DOCX) [file pone.0135688.s006.docx]

**Table S5*.*** Bonferroni-corrected post-hoc tests of within-treatment mean nest recognition attempts by *Megachile rotundata* females to enter her own nest before and after fungicide and adjuvant sprays in a cage study in North Logan, Utah in 2012.

| Effect | SE | *t* | Adj *P* |
| --- | --- | --- | --- |
| Control – Week 1 × Week 2 | 0.214 | 3.67 | <0.0001 |
| Control – Week 1 × Week 3 | 0.288 | 6.84 | <0.0001 |
| Control – Week 2 × Week 3 | 0.135 | 5.95 | <0.0001 |
| ADJ – Week 1 × Week 2 | 0.198 | 2.99 | 0.003 |
| ADJ – Week 1 × Week 3 | 0.241 | 7.62 | <0.0001 |
| ADJ – Week 2 × Week 3 | 0.310 | 10.32 | <0.0001 |
| PRI – Week 1 × Week 2 | 0.145 | 15.55 | <0.0001 |
| PRI – Week 1 × Week 3 | 0.288 | 13.10 | <0.0001 |
| PRI – Week 2 × Week 3 | 0.210 | 9.24 | <0.0001 |
| PRI+ADJ – Week 1 × Week 2 | 0.279 | 14.31 | <0.0001 |
| PRI+ADJ – Week 1 × Week 3 | 0.175 | 12.32 | <0.0001 |
| PRI+ADJ – Week 2 × Week 3 | 0.166 | 8.78 | <0.0001 |
