# Supplementary material for: Effects of Fungicide and Adjuvant Sprays on Nesting Behavior in Two Managed Solitary Bees, Osmia lignaria and Megachile rotundata
Source: PLoS One. 2015 Aug 14;10(8):e0135688. doi: 10.1371/journal.pone.0135688 (PMC4537283; doi:10.1371/journal.pone.0135688)
Supplement: S6 Table — (DOCX) [file pone.0135688.s007.docx]

**Table S6*.*** Bonferroni-corrected post-hoc tests of within-treatment mean nest recognition attempts by *Megachile rotundata* females to enter other nests before and after fungicide and adjuvant sprays in a cage study in North Logan, Utah in 2012.

| Effect | SE | *t* | Adj *P* |
| --- | --- | --- | --- |
| Control – Week 1 × Week 3 | 0.225 | 5.54 | <0.0001 |
| Control – Week 2 × Week 3 | 0.238 | 6.22 | <0.0001 |
| ADJ – Week 1 × Week 2 | 0.149 | 3.11 | 0.001 |
| ADJ – Week 1 × Week 3 | 0.178 | 8.10 | <0.0001 |
| ADJ – Week 2 × Week 3 | 0.222 | 5.77 | <0.0001 |
| PRI – Week 1 × Week 2 | 0.294 | 11.34 | <0.0001 |
| PRI – Week 1 × Week 3 | 0.237 | 15.54 | <0.0001 |
| PRI – Week 2 × Week 3 | 0.196 | 10.57 | <0.0001 |
| PRI+ADJ – Week 1 × Week 2 | 0.301 | 8.61 | <0.0001 |
| PRI+ADJ – Week 1 × Week 3 | 0.253 | 14.40 | <0.0001 |
| PRI+ADJ – Week 2 × Week 3 | 0.285 | 7.99 | <0.0001 |
